# Prolonged exposure to simulated microgravity promotes stemness impairing morphological, metabolic and migratory profile of pancreatic cancer cells: a comprehensive proteomic, lipidomic and transcriptomic analysis

**DOI:** 10.1007/s00018-022-04243-z

**Published:** 2022-04-07

**Authors:** Maria Angela Masini, Valentina Bonetto, Marcello Manfredi, Anna Pastò, Elettra Barberis, Sara Timo, Virginia Vita Vanella, Elisa Robotti, Francesca Masetto, Francesca Andreoli, Alessandra Fiore, Sara Tavella, Antonio Sica, Massimo Donadelli, Emilio Marengo

**Affiliations:** 1 Department of Sciences and Innovation Technologies (DISIT), University of Eastern Piedmont, Alessandria, Italy; 2 Department of Translational Medicine (DIMET), University of Eastern Piedmont, Novara, Italy; 3 Humanitas Clinical and Research Center, IRCCS, Rozzano, Milan Italy; 4 Department of Neurosciences, Biomedicine and Movement Sciences (DNBM), University of Verona, Verona, Italy; 5 Department of Experimental Medicine (DIMES), University of Genova, Genova, Italy; 6 Department of Pharmaceutical Sciences (DSF), University of Eastern Piedmont ‘A. Avogadro’, Novara, Italy; 7 IRCCS Ospedale Policlinico San Martino, Genova, Italy; 8 ISALIT, Novara, Italy; 9 CAAD, Center for Autoimmune and Allergic Disease, University of Eastern Piedmont, Novara, Italy; 10 School of Cancer & Pharmaceutical Sciences New Hunt’s House, Guy’s Campus, King’s College London, SE1 UL London, United Kingdom

**Keywords:** Microgravity, Cancer stem cells, Metabolism, Proteomic, Lipidomic

## Abstract

**Background:**

The impact of the absence of gravity on cancer cells is of great interest, especially today that space is more accessible than ever. Despite advances, few and contradictory data are available mainly due to different setup, experimental design and time point analyzed.

**Methods:**

Exploiting a Random Positioning Machine, we dissected the effects of long-term exposure to simulated microgravity (SMG) on pancreatic cancer cells performing proteomic, lipidomic and transcriptomic analysis at 1, 7 and 9 days.

**Results:**

Our results indicated that SMG affects cellular morphology through a time-dependent activation of Actin-based motility via Rho and Cdc42 pathways leading to actin rearrangement, formation of 3D spheroids and enhancement of epithelial-to-mesenchymal transition. Bioinformatic analysis reveals that SMG may activates ERK5/NF-κB/IL-8 axis that triggers the expansion of cancer stem cells with an increased migratory capability. These cells, to remediate energy stress and apoptosis activation, undergo a metabolic reprogramming orchestrated by HIF-1α and PI3K/Akt pathways that upregulate glycolysis and impair β-oxidation, suggesting a de novo synthesis of triglycerides for the membrane lipid bilayer formation.

**Conclusions:**

SMG revolutionizes tumor cell behavior and metabolism leading to the acquisition of an aggressive and metastatic stem cell-like phenotype. These results dissect the time-dependent cellular alterations induced by SMG and pave the base for altered gravity conditions as new anti-cancer technology.

**Supplementary Information:**

The online version contains supplementary material available at 10.1007/s00018-022-04243-z.

## Introduction

The force of gravity affects all living beings on Earth. From 1969, when men stepped on the moon, the importance of gravity was clear. During their stay in space, astronauts showed many physiological changes mainly impairing the musculoskeletal and cardiovascular system [1], due to the impact of reduced gravity on cellular properties including morphology, metabolism, and proliferation. In the last years, it has grown a huge interest in the effects of the absence of gravity on tumor cells in order to exploit microgravity for the development of novel anti-cancer therapies. In gravity-altered conditions, cancer cells behave differently from healthy ones: some processes, including apoptosis, adhesion, proliferation, and migration, are differently activated in cancer cells [2, 3]. Indeed, it was demonstrated that microgravity induces apoptosis [3] through the up-regulation of the pro-apoptotic factors Bax, PARP and p53, and inhibition of proliferation of cancer cells, but not of healthy cells. In addition, tumor cells are subjected to profound morphological rearrangement, linked to F-actin accumulation [4]. We and others [5, 6] proved that cytoskeleton, in particular the microfilamentous and microtubular components, became disorganized after a very short time of exposure to microgravity. This alteration affected many genes involved in the regulation of cancer cell proliferation, susceptibility to drugs [7], DNA repair (e.g. PARP), cell damage and invasion [2, 8]. However, to date the results of microgravity effects on cancer cells are contradictory mainly due to different setups, experimental designs and time points analyzed.

Given the difficulty of exposing cells and organisms to the real absence of gravity, many experiments are conducted on Earth, using tools that simulate the reduction of the gravity vector. The 3-Dimensional clinostat, Random Positioning Machine (RPM) is one of the most useful devices in this perspective. It simulates some of the physical effects of spaceflight by providing a gravity vector average reduction (down to 10^−6^ g) of the apparent force of gravity, without generating significant shear forces [9].

Exploiting the RPM tool, we decided to perform a comprehensive analysis of the long-term effects of simulated microgravity (SMG) on pancreatic ductal adenocarcinoma (PDAC) cells. Despite its incisiveness, PDAC has not been examined so far in microgravity conditions. This neoplasia is one of the most severe malignancies, representing the 4^th^-5^th^ cause of death from cancer in the western world. The therapeutic option currently available for PDAC is surgery, alone or in combination with radio or chemotherapy; however, only 8% of patients remain alive 5 years after diagnosis due to late diagnosis and/or resistance to the treatments. It is largely accepted that resistance to conventional anti-cancer treatments is due to a small cell subset called cancer stem cells (CSCs). CSCs are the main orchestrators of tumor establishment, metastatic progression and relapse. Already identified in most of the neoplastic tissues, differently from other tumor cells, CSCs are endowed with great plasticity, capability to enter quiescence and survive to microenvironment changes adapting metabolism, energy machinery and proliferation.

Performing a comprehensive transcriptomic, proteomic and lipidomic analysis in PDAC cells exposed for different time points to SMG, we demonstrated that microgravity induces cell transformation towards the acquisition of cancer stem cell-like features, leading to a more aggressive and metastatic phenotype.

## Materials and methods

### Cell cultures

Human ductal pancreatic adenocarcinoma cell lines PaCa-44 and CFPAC-1 were grown in RPMI-1640 medium supplemented with 10% fetal bovine serum (FBS), 1% penicillin and streptomycin (PS). Human ductal pancreatic adenocarcinoma cell line AsPC-1 was grown in DMEM medium supplemented with 10% fetal bovine serum (FBS), 1% penicillin and streptomycin (PS). Cells were plated at the final concentration of 5 × 10^5^ cells/ml on flask, Petri dish or flask on slide, according to the experimental design, and maintained at 37 °C with 5% CO_2_.

For simulated microgravity (SMG) cell culture 5 × 10^5^ cells/ml were seeded in slide flasks and subsequently fitted onto a Random Positioning Machine (RPM, Dutch Space, NL), where they were kept under continuous rotation at 56 deg/s and at the temperature of 37 °C, for 1, 7 or 9 days. To avoid air bubbles formation and to ensure a minimization of turbulence and shear stress, flasks were completely filled with medium [9]. Flasks were placed close to the support center to minimize any centrifugal acceleration, using “real random mode”. In this manner, speed and direction of the two rotating frames, changed randomly under the control of a software [10].

For 3D culture, PaCa-44 cells were seeded onto U-bottom low adhesion 96 well-plates (Greiner Bio-One) at a concentration of 5 × 10^4^ cells/well in 100 μl RPMI-1640 complete medium supplemented with 0,5% methyl-cellulose (spheroid-forming medium). Spheroids were grown at 37 °C and 5% CO_2_. Cells subjected to SMG were compared to two different control groups: frame controls and ground controls. Frame control cells (F, 1xg) were placed on the frame supporting the RPM in order to expose them to any vibration eventually produced and transmitted by the rotating machinery to the supporting structure. Ground control cells (1 g) both in spheroid-forming culture condition (Spheroid 1 g) and normal adhesion (Adhesion) were kept in an incubator at 37 °C and 5% CO_2_. Controls were analyzed at the same time points of SMG-cells.

### Spheroid staining and immunofluorescence

Spheroids were fixed in 4% PFA, dehydrated and embedded in London Resin White. Spheroids’ Sects. (1 μm thickness) were stained with toluidine blue and morphologically analyzed using an optic microscope (Zeiss Axiovert 100 M, Oberkochen, Germany).

For Calcein/PI staining, unfixed spheroids were stained with Calcein-AM (1 mM) for 20 min, followed by staining with PI (0.4 mg/ml) for 5 min. Then, spheroids were washed twice in PBS solution (0.1 M, pH 7.2) and observed at a fluorescent microscope (Axiovert 100 M, Zeiss). Fluorescent images were captured using AxioObserver Z2 inverted microscope (Zeiss), with Apotome2 system (Zeiss) and ZEN Blue 2.6 image acquisition software (Zeiss).

### Proteomic analysis

At different time points (according to the experimental setting), the cells were collected, washed in PBS and resuspended in RIPA buffer (Thermo Fisher Scientific, Waltham, MA, USA) supplemented with protease inhibitors cocktail 1X (Roche, Basilea, Switzerland). To increase yields, cells were sonicated twice for 10 min; the lysate was gently mixed for 15 min on ice and then centrifuge at 14,000×*g* for 15 min at 4 °C to pellet the cell debris. Protein concentration was measured with BCA Protein Assay (Thermo Fisher Scientific) using bovine serum albumin as a standard. The cell lysates were reduced using 2.5 μL of dithiothreitol (200 mM DTT stock solution) (Sigma-Aldrich, St.Louis, MO, USA) at 90 °C for 20 min, and alkylated with 10 μl of Cysteine Blocking Reagent (Iodoacetamide, IAM, 200 mM Sigma-Aldrich) for 1 h at room temperature in the dark. Trypsin (Promega, Sequence Grade) was added and digestion was performed overnight at 37 °C. Then, the peptides digests were desalinated on the Discovery® DSC-18 solid phase extraction (SPE) 96-well Plate (25 mg/well) (Sigma-Aldrich) and the samples were ready for the analysis.

LC–MS/MS analyses were performed using a micro-LC Eksigent Technologies (Dublin, CA, USA) system with a stationary phase of a Halo Fused C18 column (0.5 × 100 mm, 2.7 μm; Eksigent Technologies). The mobile phase was a mixture of 0.1% (v/v) formic acid in water (A) and 0.1% (v/v) formic acid in acetonitrile (B), eluting at a flow-rate of 15.0μL min − 1 at an increasing concentration of solvent B from 2 to 40% in 30 min. The samples used to generate the SWATH-MS (Sequential window acquisition of all theoretical mass spectra) spectral library were subjected to the traditional data-dependent acquisition (DDA): the mass spectrometer analysis was performed using a mass range of 100–1500 Da (TOF scan with an accumulation time of 0.25 s), followed by a MS/MS product ion scan from 200 to 1250 Da (accumulation time of 5.0 ms) with the abundance threshold set at 30 cps (35 candidate ions can be monitored during every cycle). Samples were then subjected to cyclic data independent analysis (DIA) of the mass spectra, using a 25-Da window. A 50-ms survey scan (TOF–MS) was performed, followed by MS/MS experiments on all precursors. These MS/MS experiments were performed in a cyclic manner using an accumulation time of 40 ms per 25-Da swath (36 swaths in total) for a total cycle time of 1.5408 s. The ions were fragmented for each MS/MS experiment in the collision cell using the rolling collision energy. The MS data were acquired with Analyst TF 1.7 (AB SCIEX, Concord, Canada). Three instrumental replicates for each sample were subjected to the DIA analysis.

The mass spectrometry files were searched using Protein Pilot (AB SCIEX) and Mascot (Matrix Science Inc., Boston, USA). Samples were input in the Protein Pilot software v. 4.2 (AB SCIEX), with the following parameters: cysteine alkylation, digestion by trypsin, no special factors and False Discovery Rate at 1%. The UniProt Swiss-Prot reviewed database containing human proteins (version 12/10/2018, containing 48,561 sequence entries). The Mascot search was performed on Mascot v. 2.4, the digestion enzyme selected was trypsin, with 2 missed cleavages and a search tolerance of 50 ppm was specified for the peptide mass tolerance, and 0.1 Da for the MS/MS tolerance. The charges of the peptides to search for were set to 2 + , 3 + and 4 + , and the search was set on monoisotopic mass. The instrument was set to ESI-QUAD-TOF and the following modifications were specified for the search: carbamidomethyl cysteine as fixed modification and oxidized methionine as variable modification [11, 12].

The quantification was performed by integrating the extracted ion chromatogram of all the unique ions for a given peptide. The quantification was carried out with PeakView 2.0 and MarkerView 1.2. (AB SCIEX). Six peptides per protein and six transitions per peptide were extracted from the SWATH files. Shared peptides were excluded as well as peptides with modifications. Peptides with FDR lower than 1.0% were exported in MarkerView for the t-test.

Ingenuity Pathways Analysis (IPA) software (Qiagen, Redwood City, CA, USA) and STRING software (www.stringdb.org), were used for bioinformatics analysis [13].

### Lipidomic analysis

Cells were extracted using a solution 75:15 IPA/H_2_O, after the addition of 100μL of MeOH 5% deuterated standard (Splash Lipidomix®). Then the samples were vortexed for 30 s, sonicated for 2 min and vortexed again for 30 s and then they were incubated for 30 min at 4 °C, under a gentle, constant shaking. Another 30 min were used to keep the sample rest in ice. To remove debris and other impurities, the samples were centrifuged for 10 min at 3500*g* at 4 °C. 1 mL of supernatant was collected and dried using a SpeedVac. The dried samples were reconstituted in 100μL of MeOH containing the internal standard CUDA (12.5 ng/mL).

For the analysis of the reconstituted lipids a UHPLC Vanquish system (Thermo Scientific, Rodano, Italy) coupled with an Orbitrap Q-Exactive Plus (Thermo Scientific) was used. A reverse phase column was used for the separation of lipids (Hypersil Gold™ 150 × 2.1 mm, particle size 1.9 µm), the column was maintained at 45 °C at a flow rate of 0.260 mL/min. Mobile phase A for the ESI positive mode consisted of acetonitrile/water 60:40 (v/v) while B was isopropanol/acetonitrile 90:10 (v/v) both modified with ammonium formate (10 mM) and 0.1% formic acid while in the negative ESI mode the same organic solvents and same proportioning were used, except for the use of ammonium acetate (10 mM) as mobile-phase modifier instead of ammonium formate. The gradient used was as follows: 0–2 min from 30 to 43% B, 2–2.1 min from 43 to 55% B, 2.1–12 min from 55 to 65% B, 12–18 min from 65 to 85% B, 18–20 min from 85 to 100% B; 100% B was kept for 5 min and then the column was allowed to re-equilibrate at 30% B for another 5 min. The total run time was 30 min.

Mass spectrometry analysis was performed in both positive and negative ion mode. The source voltage was maintained at 3.5 kV in the positive ion mode and 2.8 kV in the negative ion mode. All other interface settings were identical for the two types of analysis. The capillary temperature, sheath gas flow, and auxiliary gas flow were set at 320 °C, 40 arb, and 3 arb respectively. S-lens was settled at 50 rf. Data were collected in a data-dependent (ddMS2) top 10 scan mode. Survey full-scan MS spectra (mass range m/z 80 to 1200) were acquired with resolution *R* = 70,000 and AGC target 1 × 106. MS/MS fragmentation was performed using high-energy c-trap dissociation (HCD) with resolution *R* = 17,500 and AGC target 1 × 105. The stepped normalized collision energy (NCE) was set to 15, 30, and 45, respectively. The injection volume was 3µL. Lockmass and regular inter-run calibrations were used for accurate mass-based analysis. An exclusion list for background ions was generated analysing the same procedural blank sample, both for the positive and negative ESI mode.

The acquired raw data from the untargeted analysis were processed using MSDIAL software (Yokohama City, Kanagawa, Japan), version 4.24. This included the detection of peaks, MS2 data deconvolution, compound identification, and the alignment of peaks through all the samples.

For identification a cut off value of 85% was selected: this value is based on 6 different similarity scores: 1 for retention time, 1 for m/z 1 for isotopic pattern, and 3 for MS/MS (dot product, dot product reversed and presence). The dataset containing m/z values, retention time, peak area, and annotation from the aligned files were exported as an Excel file and manually checked in order to eliminate signals from blanks or wrong records.

For quantification, the peak area for different detected molecular species for each particular lipid was combined (e.g., [M + NH4] + & [M + Na] + for TG) followed by normalization using the deuterated internal standard for each lipid class. In order to obtain an estimated concentration expressed in ug/mL the normalized areas were multiplied by the concentration of the internal standard. An in-house library of standards was also used for lipids identification. MetaboAnalyst 4.0 software (www.metaboanalyst.org) was used for statistical analysis [14].

### Spheroid migration assay

96-well flat-bottomed plates were coated with 50ul/well of gelatin 0.1% (v/v) in sterile ddH2O. The plate was incubated at room temperature for 2 h; then the residual of gelatin was aspirated. The wells were washed twice with PBS and 100ul of 1% (w/v) BSA (Sigma Aldrich) in PBS was added for 1 h at room temperature. Residual BSA was aspirate and one single spheroid was plated in each well in 200ul of complete culture medium (RPMI-1640). The plate was then transferred for 30 min in the incubator to allow spheroids to adhere to the coated surface before imaging for the *t* = 0 time point. Pictures were analyzed using Image-Pro premier software and the migration capability was evaluated as the delta volume compared to the spheroid at T0.

### FACS sorting

PaCa-44 cells were stained with Live/Dead fixable dye (1:1000; ThermoFisher) and anti-human EpCAM (1:100, BD, Franklin Lakes, NJ, USA) for 30 min at + 4 °C. After washed in MACS buffer, cells were FACS-sorted with a MoFlo Astrios Cell Sorter (Beckman Coulter, Brea, CA); the purity of the sorted populations always exceeded 90%.

### Lactate dehydrogenase (LDH) assay

To evaluate LDH enzyme activity the CyQUANT™ LDH cytotoxicity assay kit (Invitrogen, Thermo Fisher Scientific) was used according to the manufacturer’s instructions. 50 µL of supernatant was collected from cells cultured in the different culture conditions (normal adhesion, spheroid at 1 g and simulated microgravity, SMG) at the end of each experiment and dispensed into a new 96-well plate (Thermo Fisher Scientific). LDH activity was quantified with Tecan Infinite F200Pro plate reader (Tecan, Austria) at 490 nm absorption.

### Cell proliferation MTT assay

10^5^ cells were seeded in 100ul of complete culture medium (RPMI-1640) in 96 well flat-bottomed plates. For proliferation analysis in PaCa-44 cells grown in adhesion culture condition, the cells were allowed to adhere to the plate o/n in the incubator. 10 μl of MTT solution (final concentration 0.5 mg/ml) was added to each well and the plate transferred for 4 h in the incubator. The reaction was then stopped adding 100 μl/well of solubilization solution (12 M HCl, isopropanol and TritonX 10%). After 15 min in the incubator, the absorbance of the samples was measured using a microplate reader at the wavelength of 570 nm. Results were presented as delta absorbance between the samples and the blank condition.

### TUNEL assay

Apoptosis was evaluated using the In Situ Cell Death Detection Kit, Fluorescein (Roche, Lewes, UK) in the spheroids obtained in SMG. Spheroids were fixed in PFA 4%, embedded in Technovit 8100 resin (BioOptica, Milan, Italy) and sectioned. Sections were permeabilized with 0.1% Triton X-100 in 0.1% sodium citrate and exposed to TUNEL reaction mixture for 1 h at 37 °C, in a humidified atmosphere, protected from light. After PBS washes, sections were analyzed with AxioObserver Z2 inverted microscope (Zeiss), with Apotome2 system (Zeiss) and ZEN Blue 2.6 image acquisition software (Zeiss).

#### Western Blot analysis

After 9 days of maintenance in the different culture conditions (adhesion, spheroid at 1 g and simulated microgravity), cells were collected and lysed with RIPA buffer supplemented with protease and phosphatase inhibitors (PPC1010, Sigma-Aldrich, Milan, Italy). The protein concentration was determined using BCA protein assay (71,285, Sigma-Aldrich, Milan, Italy) and equal amounts of protein were loaded on NuPAGE™ 4–12% Bis–Tris protein precast polyacrylamide gels (Invitrogen, Thermo Fisher Scientific) in denaturing and reducing conditions. After transfection into nitrocellulose membranes (Perkin Elmer), membranes were saturated with 5% non-fat milk in TBS- 0.1% Tween 20 buffer, and hybridized overnight at 4 °C with the following primary anti-human antibodies: rabbit anti-N-cadherin (1:3000**,** Genetex, Irvine, CA), rabbit anti-E-cadherin (1:3000**,** Genetex), rabbit anti-NF-kB p50 (1:1000, Santa Cruz), rabbit anti-NF-kB p65 (1:1000, Santa Cruz), rabbit anti-phopsho-NF-kB p65 (Ser536, 1:1000, Cell Signaling, Boston, MD), mouse anti-Epcam (1:1000, Cell Signaling), mouse anti-GAPDH (1:1000, Santa Cruz), mouse anti-α-Tubulin (1:1000, Merckmillipore), mouse anti-β-Actin (1:1000, Cell Signaling), rabbit anti-LDHA (1:2000, Cell signaling) and mouse anti-Nanog (1:1000, Biorbyt, Cambridge, UK). Membranes were then washed and probed for 1 h at room temperature with HRP-conjugated secondary antibodies and developed using chemiluminescence reagents (SuperSignal™ West Pico PLUS Chemiluminescent Substrate, 34,580, Thermo Fisher Scientific). Membranes were scanned using a ChemiDoc™ XRS Imaging System (Bio-Rad, Hercules, CA) and bands were quantified using NIH Image J software (Version 1.53f51, National Institutes of Health (NIH), Bethesda, MA, USA).

### RNA extraction and qRT-PCR

Total RNA was extracted, using TRIzol Reagent (Thermo Fisher Scientific), from PaCa-44 cells cultured under all the different culture conditions. 1 μg of RNA was reverse transcribed using first-strand cDNA synthesis. Real-time qPCR quantification was performed in triplicate samples by SYBR-Green detection chemistry with GoTaq qPCR Master Mix (Promega, Madison, WI, USA) with a QuantStudio 3 Real-Time PCR System (Thermo Fisher Scientific, USA). The primers used are listed in Table 1. The cycling conditions used were: 95 °C for 10 min, 40 cycles at 95 °C for 15 s, 60 °C for 1 min, 95 °C for 15 s, and 60 °C for 15 s. The average of the cycle threshold of each triplicate was analyzed according to the 2^−ΔΔCt^ method. 18S gene expression was used as endogenous control to standardize mRNA expression.

**Table 1:** Sequences of primers used for qPCR analysis

GENES	FORWARD PRIMER SEQUENCE	REVERSE PRIMER SEQUENCE
CDH1	5′-GACACCAACGATAATCCTCCGA-3′	5’-GGCACCTGACCCTTGTACGT-3’
ZEB1	5′-GTTACCAGGGAGGAGCAGTGAAA-3′	5′-GACAGCAGTGTCTTGTTGTTGTAGAAA-3′
EpCAM	5'-CCGCTGCGAGGACGTAGA-3'	5'-TGTTGGCTGCGTCTCATCAAACC-3'
CASP 3	5’-CTGGTTTTCGGTGGGTGT-3’	5’-CACTGAGTTTTCAGTGTTCTCCA-3’
CASP 8	5’-CAGCAGCCTTGAAGGAAGTC-3’	5’-CGAGATTGTCATTACCCCACA-3’
CASP 9	5’-CCCAAGCTCTTTTTCATCCA-3’	5’-AGTGGAGGCCACCTCAAAC-3’
BAD	5’-ACCAGCAGCAGCCATCAT-3’	5’-GGTAGGAGCTGTGGCGACT-3’
TRAIL	5’-CCTCAGAGAGTAGCAGCTCACA-3’	5’-CAGAGCCTTTTCATTCTTGGA-3’
BAX	5’-CAAGACCAGGGTGGTTGG-3’	5’-CACTCCCGCCACAAAGAT-3’
N-CDH	5’-CCTCCAGAGTTTACTGCCATGAC-3’	5’-GTAGGATCTCCGCCACTGATTC-3’
OCT4	5′-GACAG GGGGAGGGGAGGAGCTAGG-3′	5′-CTTCCCTCCAACCAGTTGCCCCAAAC-3
NANOG	5′-AGTCCCAAAGGCAAACAACCCACTTC-3′	5′-TGCTGGAGGCTGAGGTATTTCTGTCTC-3′
SOX2	5′-GGGAAATGGGAGGGGTGCAAAAGAGG-3′	5′-TTGCGTGAGTGTGGATGGGATTGGTG-3′
18S	5’-ACTTTCGATGGTAGTCGCCGT-3’	5’-CCTTGGATGTGGTAGCCGTTT-3’
GAPDH	5′-ATCAGCAATGCCTCCTGCAC-3′	5′-TGGTCATGAGTCCTTCCACG-3′

### Statistical analysis

Values were expressed as mean ± s.e.m. Either one-way ANOVA or two-tailed, unpaired Student’s *t*-test was used to compare unmatched groups with Gaussian distribution. A Mann–Whitney *U*-test was used in cases of non-Gaussian distribution. Two-way ANOVA corrected for multiple comparison tests was used to take into account multiple comparisons. P ≤ 0.05 was considered statistically significant. Statistical analyses were performed with GraphPad Prism v.7 GraphPad Software.

## Results

### Simulated microgravity alters the proteomic profile of PaCa-44 cells in a time-dependent manner

**Fig. 1:**
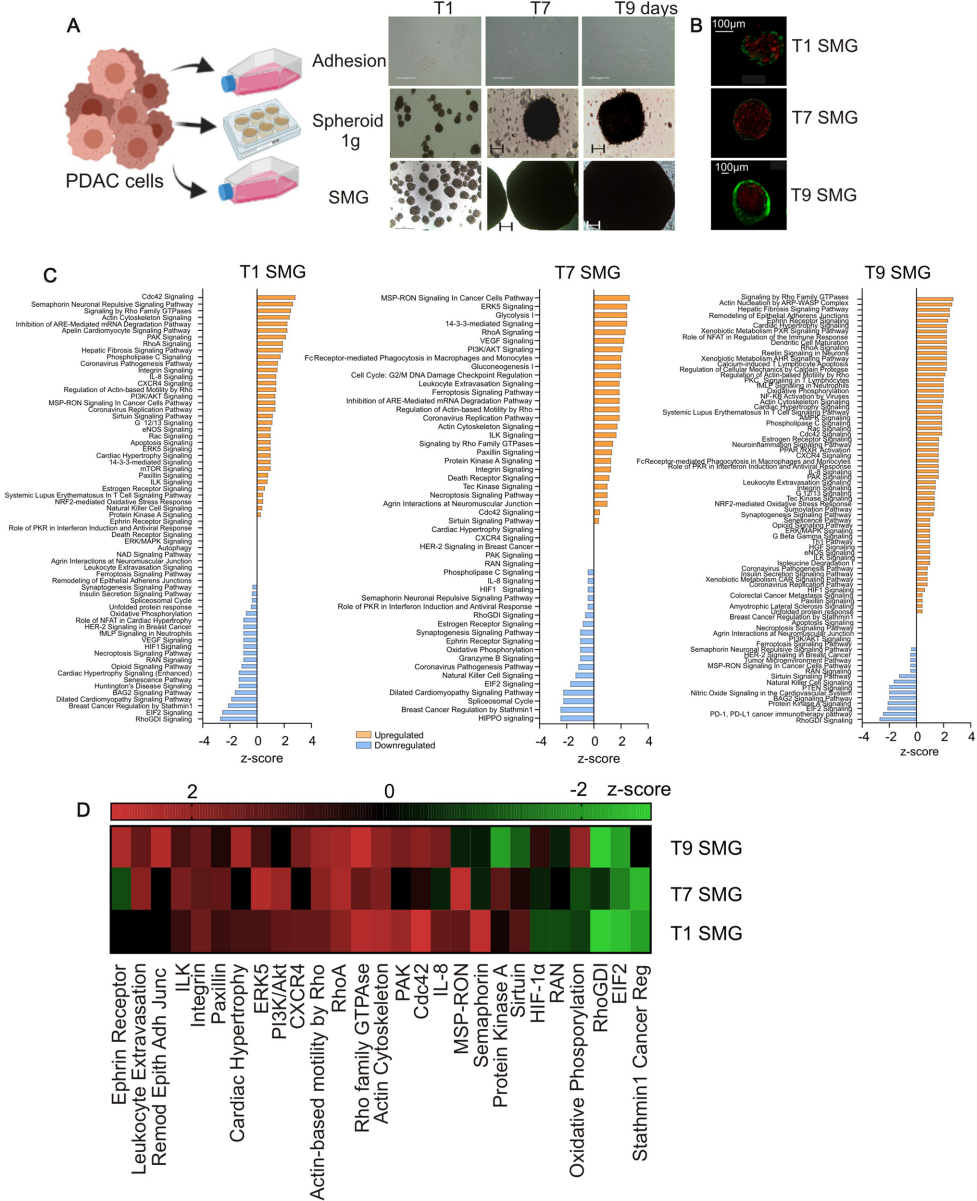
Simulated microgravity (SMG) alters the proteomic profile of PaCa-44 cells in a time-dependent manner. **A** PaCa-44 cells were cultured in adhesion condition at 1 g (Adhesion) or in SMG, and in spheroid-forming culture condition at normal gravity (Spheroid 1 g). Representative pictures acquired at 1, 7 and 9 days of culture (T1, T7 and T9, respectively); scale bar 400 μm for Adhesion, 200 μm for Spheroids 1 g and SMG pictures. **B** Calcein/PI staining on spheroids obtained after 1, 7 and 9 days in SMG culture conditions (T1, T7 and T9 SMG, respectively). Scale bar 100 μm. **C** Canonical pathways modulated by differentially expressed proteins after 1, 7 or 9 days of SMG (T1, T7 and T9 SMG respectively). The pathways are indicated in the y-axis. On the x-axis, the z-score for each pathway calculated in comparison to the adhesion culture condition. Orange bars predict an overall increase in the pathway activity, while blue bars predict a decrease. **D** Heat-map of z-score of the canonical pathways modulated in all of the three different time points presented in panel C (1, 7 and 9 days in SMG)

To investigate the effects of microgravity on PDAC, PaCa-44 cells were plated in complete medium in uncoated flasks and cultured at 1 g (as control, Adhesion) or in RPM (SMG); in parallel some cells were plated in spheroid-forming medium in u-bottom low adhesion 96 well-plates at 1 g (Spheroids 1 g, Fig. 1A). While in Adhesion the cells maintained their shape along all the evaluated time points, after 24 h (T1), both in 1 g spheroid-forming and SMG conditions, the cells underwent spontaneous aggregation forming small and loose tridimensional structures (Fig. 1A). These aggregates began to form spherical-like structures with a diameter around 100 µm that grew over 1 mm from the 7th to the 9th day (T7 and T9 respectively; Fig. 1A) in SMG, whereas in normal gravity they remained around 100 µm of diameter over time (Fig. 1A). When stained with Calcein-PI, spheroids obtained from SMG culture condition revealed an inner necrotic core of dead cells surrounded by vital cells accumulated in the outer layer (Fig. 1B). Similar results were obtained in other two pancreatic cancer cell lines, CFPAC-1 and AsPC-1, maintained in the same culture conditions: 9 days of SMG exposure was associated with a morphological rearrangement of the cells that grown as compact and large 3D spheres (Suppl. Fig S1, panel A and D).

To investigate the biological alterations induced by SMG exposure, PaCa-44 cells were cultured in RPM or in normal culture conditions (Adhesion 1 g, as control) in the same plastic supports, same medium, and at the same concentration (as detailed in the Methods section). At different time points (1, 7 and 9 days) a large proteomic analysis was performed with SWATH-MS using a statistical cut-off of *p*-value < 0.05 and fold change > 1.3. Results showed that 24 h of SMG induced the modulation of 360 proteins (206 up and 154 downregulated, compared to control culture condition). On the 7th day in SMG condition, cells upregulated 128 proteins and downregulated 111 (for a total of 239 proteins), whereas on the 9th day in SMG 335 proteins were modulated, of whom 208 upregulated and 127 downregulated.

The proteomic data set, including UniProt code and fold changes, was then uploaded to Ingenuity Pathway Analysis (IPA) bioinformatics tool, to identify the canonical pathways significantly modulated. After 24 h in SMG, 34 canonical pathways were upregulated (z-score > 0) and 21 downregulated (z-score < 0; Fig. 1C, T1 SMG); 10 pathways, instead, presented a modulation of protein expression but the final z-score was 0. After 7 days, 27 pathways were upregulated (z-score > 0), 18 downregulated (z-score < 0) and 5 presented z-score = 0 (Fig. 1C, T7 SMG). On the day 9 in SMG, 59 pathways were upregulated (z-score > 0), 14 downregulated (z-score < 0) and 6 presented a z-score = 0 (Fig. 1C, T9 SMG). Evaluating only the pathways enriched at all the three time points, we discovered that most of the upregulated pathways were involved in cell cytoskeletal modification such as *Integrin signaling*, *ILK signaling*, *Regulation of actin-based motility by Rho*, *RhoA signaling*, *Signaling by Rho Family GTPase* and *Actin cytoskeleton signaling* (Fig. 1D). These results clearly support the effect of SMG in cellular reorganization.

### Exposure to simulated microgravity is associated with cytoskeletal reorganization and increased migration capability

**Fig. 2:**
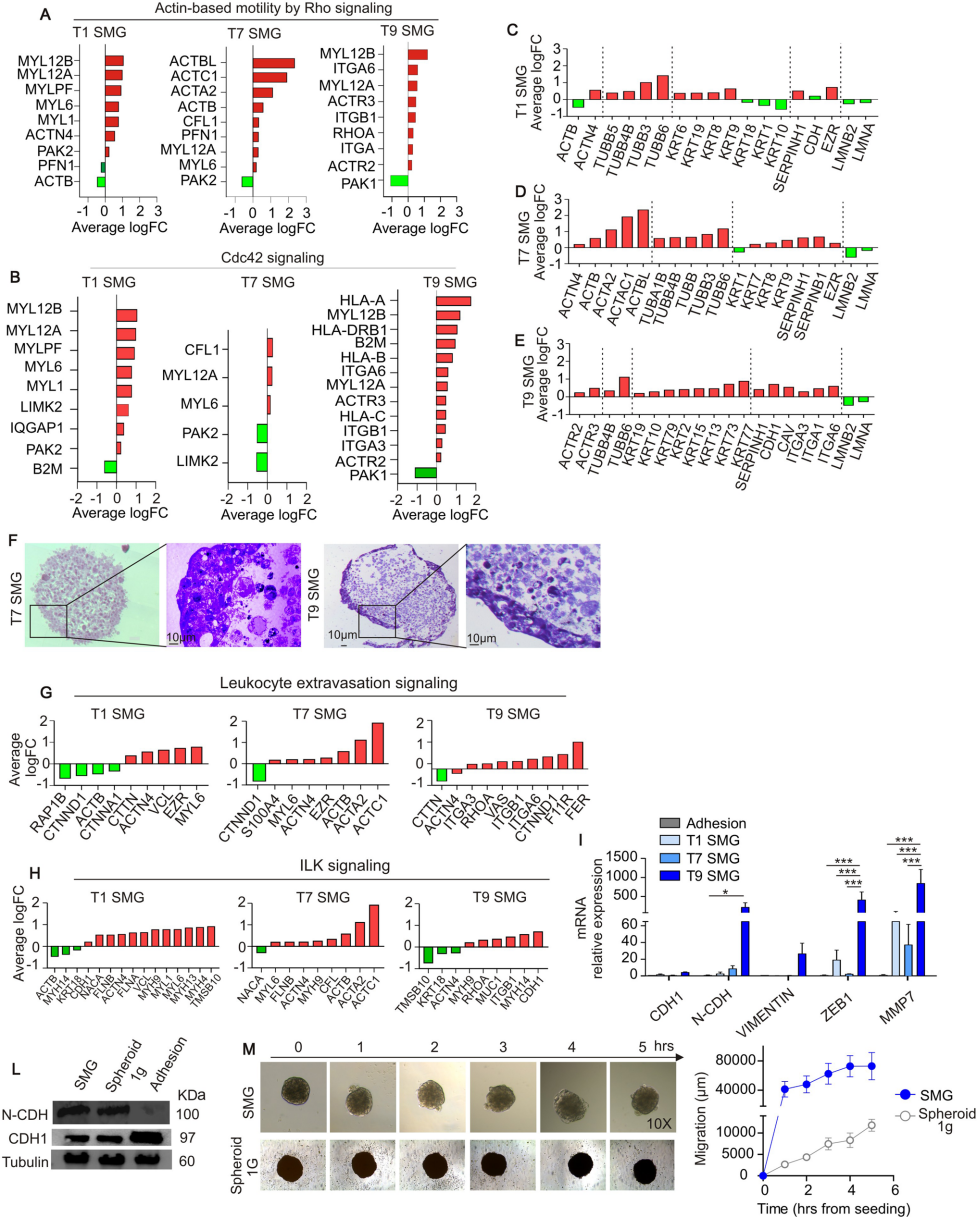
Exposure to simulated microgravity (SMG) is associated with cytoskeletal reorganization and increased migration capability. **A–B** Average logarithmic fold change of proteins involved in the Actin-based motility by Rho signaling (**A**) and Cdc42 signaling (B) evaluated in PaCa-44 cells maintained in SMG for 1, 7 or 9 days (T1, T7 and T9 SMG, respectively); in green proteins downregulated while in red proteins upregulated compared to adhesion culture conditions. **C–E** Average logarithmic fold change of proteins involved in the cytoskeletal and nuclear reorganization, evaluated in PaCa-44 cells maintained in SMG for 1 (**C**), 7 (**D**) or 9 (**E)** days; in green proteins downregulated while in red proteins upregulated compared to adhesion culture conditions at 1 g. **F** Representative pictures of Toluidine blue staining in semithin sections of spheroids obtained after 7 or 9 days in simulated microgravity (SMG); scale bar 10 μm. **G–H** Average logarithmic fold change of proteins involved in the leukocyte extravasation (**G**) and ILK (**H**) signaling evaluated in PaCa-44 cells maintained in SMG for 1 (**A**), 7 (**B**) or 9 (**C**) days (T1, T7 and T9 SMG, respectively); in green proteins downregulated while in red proteins upregulated compared to adhesion culture conditions. **I** qRT-PCR analysis of EMT-associated markers in PaCa-44 cells maintained in normal culture condition (Adhesion) or in simulated microgravity (SMG) for 1, 7 or 9 days (T1, T7 and T9 SMG, respectively). Data are expressed as mean ± sem of n = 3 replicates. *0.05, ***0.0002 and **** < 0.0001. **L** Western blot analysis of CDH-1 and N-CDH1 on PaCa-44 cells maintained in normal culture condition (Adhesion), spheroid-forming condition at 1 g (Spheroid 1 g) or in simulated microgravity (SMG) for 9 days; Tubulin is used as housekeeping. **M** Migration capability of spheroids collected on the 9th day from SMG culture or 1 g spheroid culture (Spheroid 1 g). On the left representative pictures of spheres at the different time points after seeding; on the right the graph with the mean ± sem migration calculated as spheres’ circumference at each time point compared to T0 (Delta)

One of the protein pathways most altered by SMG exposure is the “*Regulation of actin-based motility by Rho*”. RhoA signaling is involved in cytoskeleton reorganization, which induces actin migration toward the external plasma membrane where it participates in the formation of membrane blebs, thus promoting cell migration [15]. Proteomic analysis indicated a time-dependent upregulation of the pathway, from 24 h up to 9 days in SMG (Fig. 2A). Cdc42 signaling, which belongs to the Rho family of GTPase, contributes to the cytoskeletal organization throughout the perturbation of actin polymerization, as well as in the formation of actin bundle-containing filopodia at the cellular surface. Our results indicated that, as for RhoA, Cdc42 pathway was upregulated by SMG at all the 3 time points evaluated (Fig. 2B).

After 24 h in SMG, tubulin expression was upregulated (Fig. 2C); actin upregulation, instead, was evident only at longer exposure time, from 7 up to 9 days, when keratin's upregulation was massive (Fig. 2D, E). Interestingly, lamin A and B (LMNA and LMNB2, respectively), involved in oxidative stress resistance [16], were downregulated by SMG already after 24 h up to 9 days (Fig. 2C–E). In addition, 9 days of SMG exposure was associated with upregulation of E-cadherin (CDH1) and integrins (ITGA3, ITGB1 and ITGA6) that play a key role in cell adhesion to the extracellular matrix (substrate). In line, at the same time point (T9), we observed an increment of cell migration from the spheroidal structures to the flask where cells adhere to the surface (data not shown). The over time upregulation of keratins and integrins could be associated with increased cell aggregation and spheroid formation. Indeed, while after 24 h cells were mainly organized in weakened aggregates, after 7 days in SMG cells formed spheroidal structures already compact that progressively grew (up to 9 days) as demonstrated by blue toluidine staining (Fig. 2F). The spheroids appeared to be constituted by an outer shell of cells exhibiting extensive cell-to-cell adhesion, and an inner necrotic core (Fig. 2F). Such morphological changes are crucial for cell migration and metastatic potential. In line, “*Leukocytes extravasation signaling*” was altered in all the evaluated time points. Interestingly, on the 1st day in SMG the pathway was inhibited (Fig. 2G, T1 SMG) while after 7 days it was activated and remained active up to the 9th day (Fig. 2G). The upregulation of *“Actin-based motility by RhoA signaling”*, “*Leukocytes extravasation signaling*” and “*ILK signaling”* at all the analyzed time points (Fig. 2H) suggests the capability of PaCa-44 cells to adhere to the endothelium and migrate in SMG conditions. To confirm the impact of SMG on cell migration, we analyzed the expression levels of genes regulating the epithelial-to-mesenchymal transition (EMT) process at 1, 7 and 9 days of SMG exposure. EMT regulates morphological changes in tumor cells inducing the acquisition of a mesenchymal phenotype, rather than epithelial, thus supporting migration and spreading of cancer cells. qRT-PCR analysis indicated increased expression of N-cadherin (N-CDH), Vimentin and Zeb1 (mesenchymal markers), and matrix-metallopeptidase-7 (MMP-7) in PaCa-44 cells under SMG, particularly evident on the 9th day (Fig. 2I), compared to cells maintained in normal gravity adhesion condition. Modulation of E-cadherin (CDH-1) and N-CDH1 expression in PaCA-44 cells after 9 days of exposure to SMG was also confirmed by Western blot analysis (Fig. 2L). Similar results were obtained in CFPAC-1 and AsPC-1 cells: both lines showed downregulation of CDH-1 (Suppl. Fig. S1, panel B and E) and upregulation of Zeb-1 and N-CDH after 9 days in SMG (Suppl. Fig. S1, panel B, C, E, F). In addition, we performed a spheroid-migration assay comparing spheroids obtained from PaCa-44 cells maintained in SMG or spheroid-culture condition at 1 g (Spheroid 1 g) for 9 days. The results showed higher migration capability of tumor cells exposed to SMG (Fig. 2M) confirming the regulation of cell mobilization mediated by altered gravity.

### Simulated microgravity triggers cancer stem cell selection

**Fig. 3:**
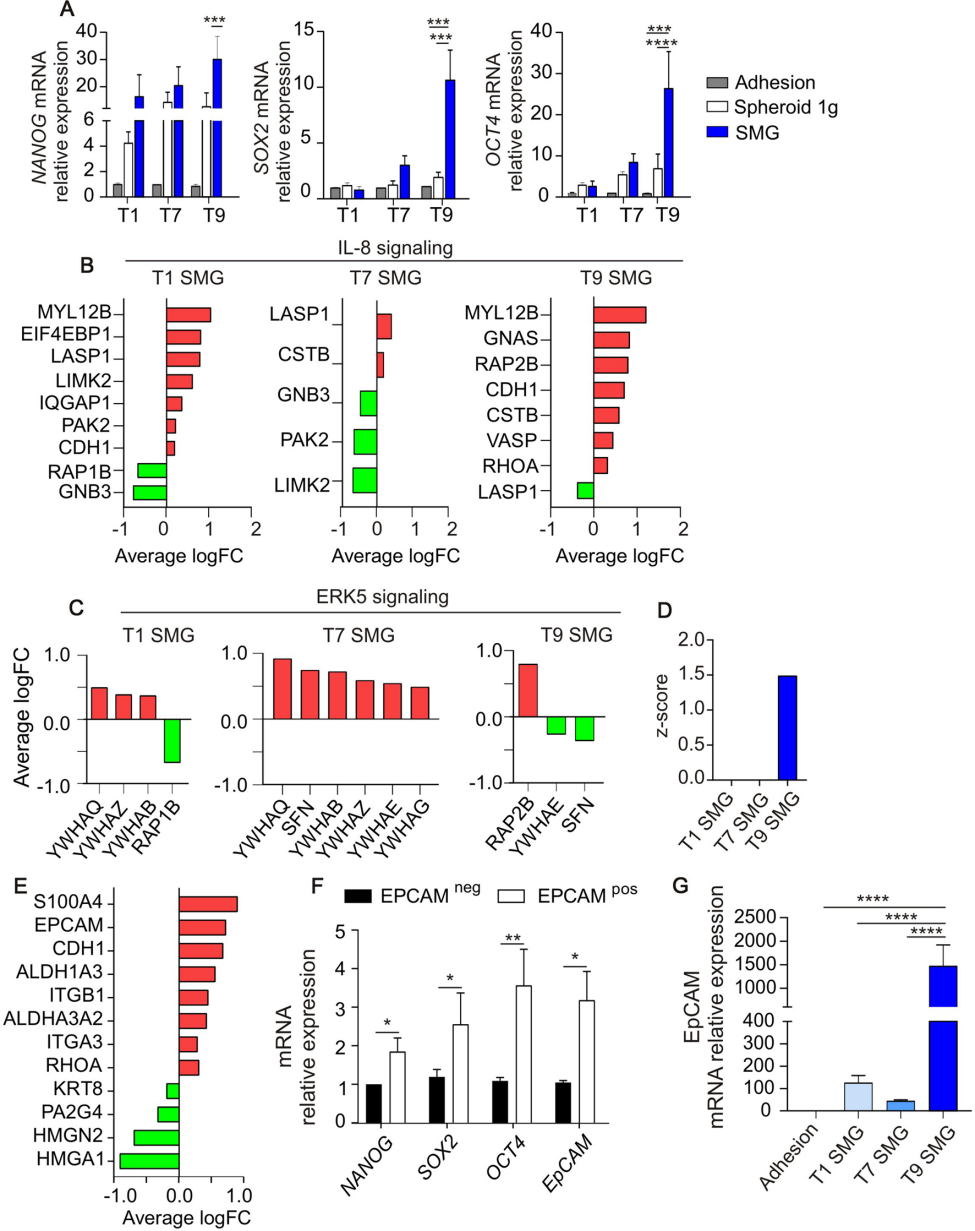
Simulated microgravity (SMG) triggers cancer stem cell selection. **A** qRT-PCR analysis of stemness-associated genes in PaCa-44 cells maintained in adhesion culture condition at normal gravity (Adhesion), altered gravity (SMG), or in spheroid-forming culture condition at normal gravity (Spheroid 1 g) for 1, 7 or 9 days. Data are expressed as mean ± sem of n = 3 replicates. * 0.05, ***0.0002 and **** < 0.0001. **B–C** Average logarithmic fold change of proteins involved in IL-8 (**B**) and ERK5 signalings (**C**) evaluated in PaCa-44 cells maintained in SMG for 1, 7 or 9 days (T1, T7 and T9 SMG, respectively); in green proteins downregulated while in red proteins upregulated, compared to adhesion culture conditions. **D** Z-score of NF-κB pathway evaluated in PaCa-44 cells exposed to SMG for 1, 7 or 9 days. **E** Average logarithmic fold change of proteins related to stemness and epithelial-to-mesenchymal transition signaling in PaCa-44 cells maintained in SMG for 9 days. **F** qRT-PCR analysis of stemness genes in PaCa-44 cells grown in normal culture condition and FACS separated according to the expression of EpCAM. **G** qRT-PCR analysis of EpCAM expression in PaCa-44 cells maintained in Adhesion culture condition at 1 g or in SMG for 1, 7 or 9 days (T1, T7 and T9 SMG, respectively). Data are expressed as mean ± sem of n = 3 replicates. *0.05; **** < 0.0001

It has been largely demonstrated that the ability to form spheroids and migrate is related to the acquisition of a stemness phenotype. To further confirm that SMG exposure induces stemness enrichment, we evaluated the expression of specific stemness-related master genes (Nanog, Sox2 and Oct4) in PaCa-44 cells maintained in the different culture conditions. As shown in Fig. 3A, 9 days in SMG induced a significantly increased expression of all the evaluated genes. These results suggest a key role of microgravity in time-dependent stemness regulation. In support, proteomic analysis indicated that IL-8 signaling, which is involved in stemness, neo-angiogenesis and EMT process regulation [17], was upregulated after 9 days in SMG (Fig. 3B). ERK5 signaling, instead, was upregulated already after 24 h in SMG (Fig. 3C). Interestingly, it has been reported that the activation of ERK5 pathway triggers the upregulation of IL-8 signaling and NF-κB transcriptional activity, suggesting an ERK5/NF-κB/IL-8 signaling axis involved in the regulation of stem cell malignancy [18]. Proteomic analysis indicated that NF-κB signaling was upregulated on the 9th day in SMG (whereas after 1 and 7 days in SMG the pathway has a *z* -score = 0; Fig. 3D). In line, western blot evaluation of p50 and p65 NF-kB family members in PaCa-44 cells showed an evident induction of p65 expression and a concomitant inhibition of p50 expression after 9 days SMG, compared to cells maintained in normal adhesion culture condition (Adhesion) or spheroid-forming culture condition (Suppl. Fig. S2).

At this time point (T9) stemness-related proteins such as EpCAM, ALDH1A3, ALDHA3A2, S100A4, ROHA, and ITGA3 (Fig. 3F) were upregulated, indicating a cellular reprogramming towards a more stem and aggressive phenotype. It has been demonstrated [19] that EpCAM is a reliable marker for the identification of CSCs in various tumor types including pancreatic cancer. Indeed, when PaCa-44 cells were FACS sorted according to the expression of EpCAM, we observed a significantly higher expression of stemness-associated master genes (Nanog, Sox2 and Oct4) in EpCAM^pos^ compared to EpCAM^neg^ cells (Fig. 3G). Analysis of expression levels of EpCAM in PaCa-44 cells subjected to SMG indicated an over time increase of this marker, supporting our findings of a CSC enrichment mediated by altered gravity force (Fig. 3H).

### Simulated microgravity strongly edits cell metabolism

**Fig. 4:**
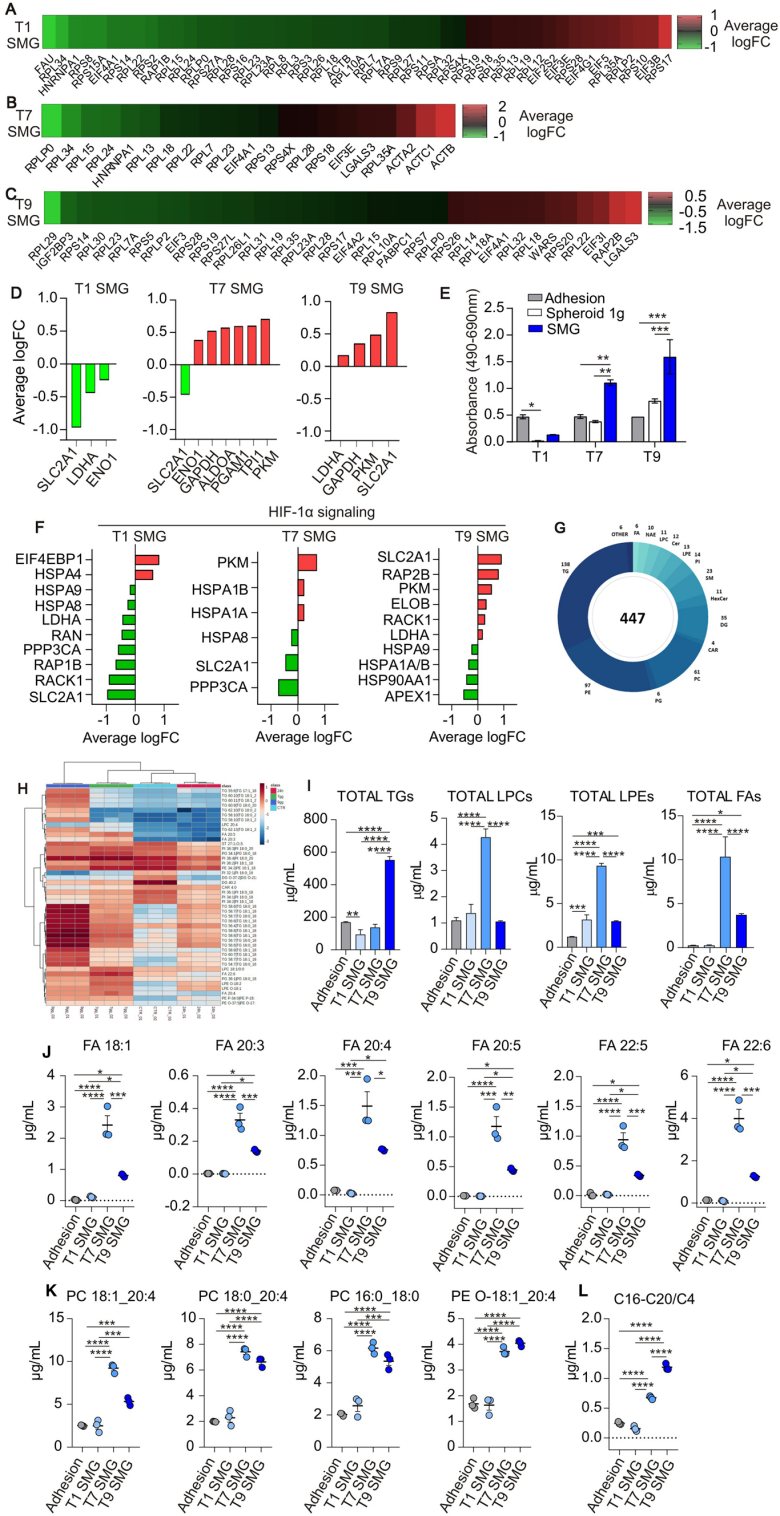
Simulated microgravity (SMG) exposure edits tumor cell metabolism. **A–C** Heat map of eiF2-related protein fold change expression, evaluated in PaCa-44 cells maintained in SMG for 1 (**A**), 7 (**B**) or 9 (**C**) days; **D** Average logarithmic fold change of proteins involved in glycolysis evaluated in PaCa-44 cells maintained in SMG for 1, 7 or 9 days; in green proteins downregulated while in red proteins upregulated compared to adhesion culture conditions. **E** Extracellular levels of LDH evaluated at 1, 7 and 9 days (T1, T7 and T9, respectively) in PaCa-44 cells maintained in adhesion or spheroid-forming culture condition at normal gravity (Adhesion or Spheroid 1 g, respectively) and in SMG. **F** Average logarithmic fold change of proteins involved in HIF-1α signaling evaluated in PaCa-44 cells maintained in SMG for 1, 7 or 9 days; in green proteins downregulated while in red proteins upregulated compared to adhesion culture conditions. **G** Donut chart of the number of identified lipids for each class. **H** Hierarchical clustering heat-map of lipid expression evaluated in PaCa-44 cells maintained in Adhesion culture condition at 1 g or in SMG for 1, 7 or 9 days. **I** Total lipid concentration for triacylglycerols (TGs), lysophosphatidylcholine (LPCs), lysophosphatidylethanolamine (LPEs) and fatty acids (FAs) evaluated in PaCa-44 cells maintained in Adhesion culture condition at 1 g or in SMG for 1, 7 or 9 days. **J–K** Lipid concentration of unsaturated fatty acids (Fas, D), phosphatidylcholines (PC) and phosphatidylethanolamines (PE, E) evaluated in PaCa-44 cells maintained in Adhesion culture condition at 1 g or in SMG. **L** Ratio of long-intermediate-chain acylcarnitines (C16-C20) to short-chain ones (C4) evaluated in PaCa-44 cells maintained in Adhesion culture condition at 1 g or in SMG for 1, 7 or 9 days. All data are expressed as mean ± sd of 3 different replicates. * 0.05, ** < 0.005; *** < 0.0005. For all the panels T1, T7 and T9 represent 1, 7 or 9 days in simulated microgravity (SMG, respectively)

The morphological rearrangement (3D spheroids) induced by SMG also affected cellular nutrition and metabolism. From the proteomic analysis, it has emerged that eIF2 signaling was the most enriched pathway modulated by SMG (Fig. 4A-C). Compared to the control, the pathway was inhibited already after 24 h (Fig. 4A) and it was maintained downregulated up to 9 days in SMG (Fig. 4B, C). eIF2 is a component of the translational machinery and plays a key role in the orchestration of protein synthesis. Repression of global protein synthesis (as in the case of eIF2 signaling inhibition) allows cells to conserve nutrients and energy for stress remediation. Indeed, it has been demonstrated that eIF2 signaling regulates autophagy and metabolism in order to maintain an energy balance in cancer cells. In line, our proteomic data revealed that glycolysis was downregulated after 24 h in SMG (as indicated by negative logFC of SLC2A1, LDH and ENO1; Fig. 4D, T1 SMG), but after 7 days was upregulated (positive logFC of ENO1, GAPDH, ALDOA, PGAM1, TPI1 and PKMI proteins; Fig. 4D, T7 SMG). The pathway remained upregulated on the 9th day (positive logFC of LDHA, GAPDH, PKM and SLC2A1 proteins; Fig. 4D, T9 SMG). In parallel, the analysis of extracellular lactate dehydrogenase (LDH) indicated a reduction of LDH levels after 1 day in SMG (compared to normal adhesion culture condition) and an increase on the 7th and 9th day in SMG (Fig. 4E) suggesting a metabolic switch of PaCa-44 cells under reduced gravity culture condition. Similar results were obtained in CFPAC-1 and AsPC-1 cells exposed to SMG. After 9 days both cell lines presented an upregulation of GAPDH and LDHA at mRNA and protein level compared to the same cells maintained in normal adhesion or spheroid-forming culturing condition in normal gravity (Suppl. Fig. S1, panel B, C, E and F).

HIF-1α, is a key regulator of cancer-related metabolic pathways, including glycolysis, gluconeogenesis, the Tricarboxylic acid (TCA) cycle, as well as metabolism of nucleotides, amino acids and lipids. According to oxygen availability, HIF-1α dynamically regulates glucose metabolism in order to reduce reactive oxygen species (ROS) accumulation, maintain redox homeostasis and sustain cell survival. Proteomic analysis indicated an inhibition of the HIF-1α signaling at 24 h and 7 days in SMG (Fig. 4F); at 9 days instead, the pathway was upregulated, in line with the over time increased dimension (and necrotic core) of spheroids. HIF activation regulates the fate of pyruvate produced by glycolysis, triggering the production of Acetyl-CoA to provide cells with substrates involved in the membrane biogenesis, orchestrated by fatty acid (FA) and phospholipids synthesis, energy production by TCA cycle and post-translational modification, such as protein acetylation. To better clarify the alteration in lipid catabolism and synthesis, lipidomic analysis was performed at the three time points of SMG exposure. 447 lipids belonging to 18 different lipid classes were identified in PaCa-44 cells (Fig. 4G), with Triacylglycerols (TGs) as the most abundant lipid class. Monoacylglycerols were not identified whereas only few diacylglycerols were detected, most likely for their re-esterification in TGs (Jones et al., 2019). The hierarchical clustering heat-map analysis (Fig. 4H) highlighted specific SMG-induced lipidomic profiles for each time point. Total TG levels decreased at 1 and 7 days in SMG, while increased at 9 days (Fig. 4I). On the contrary, both total lysophosphatidylcholines (LPCs), lysophosphatidylethanolamine (LPEs) and polyunsaturated FAs increased at 1 and 7 days in SMG to then shrank at 9 days (Fig. 4I). Interestingly, almost all the modulated FAs (FA 20:3, FA 20:4, FA 20:5, FA22:5 and FA 22:6) were polyunsaturated (Fig. 4L) that may potentially be used as signaling molecules or as substrates for obtaining energy through β-oxidation. Concomitantly, the lysophospholipids increase, at 7 days, may sustain a deep membrane phospholipid remodeling as revealed by the increment of TGs together with the reduction of LPCs, LPsE and FAs (on the 9th day). These results suggest the synthesis of novel TGs and phosphatidylcholines (PCs) at prolonged exposure to SMG (9 days), probably to sustain the request of membrane lipids. In particular, we observed an increase of PC 18:1_20:4; PC 18:0_20:4, PC O-18:1_20:4 and PE 16:0_18:0 (Fig. 4M), supporting the hypothesis of a remodeling of the cellular membrane. Furthermore, at the same time point (9 days) the marked TG increase may be explained with their packaging into lipid droplets, which have been shown to accumulate in hypoxic conditions and to be associated with higher tumor aggressiveness [20].

Concerning polyunsaturated FAs including arachidonic acid (FA 20:4) released on the 9^th^ day, we speculate a role for arachidonic acid and its bioactive derivatives in inflammation and in local signaling of CSCs. Indeed, the high degree of unsaturation makes these metabolites reactive and susceptible to oxygenation and hydrogenation reactions, suggesting metabolic flexibility that allows cells to adapt their metabolism to survive in hostile conditions, typical of CSCs [21]. Moreover, we investigated whether free FAs may be coupled with CoA to form acyl-CoA moieties that are then transferred to carnitine to generate acylcarnitine, which subsequently enters the mitochondrial matrix via the carnitine shuttle [22]. In order to understand if there is an impairment of the β-oxidation over time, we focused our attention on the acylcarnitine class. The acylcarnitine to L-carnitine ratio is recognized as a marker of carnitine deficiency and is associated with mitochondrial β-oxidation [23]. In addition, the trend of this ratio is also maintained between long-intermediate-chain acylcarnitines (C16-20) compared to short-chain ones (C4). Our data showed an increased (C16-20)/(C4) ratio over time, suggesting an impairment of the β-oxidation (Fig. 4N) [24].

**Fig. 5:**
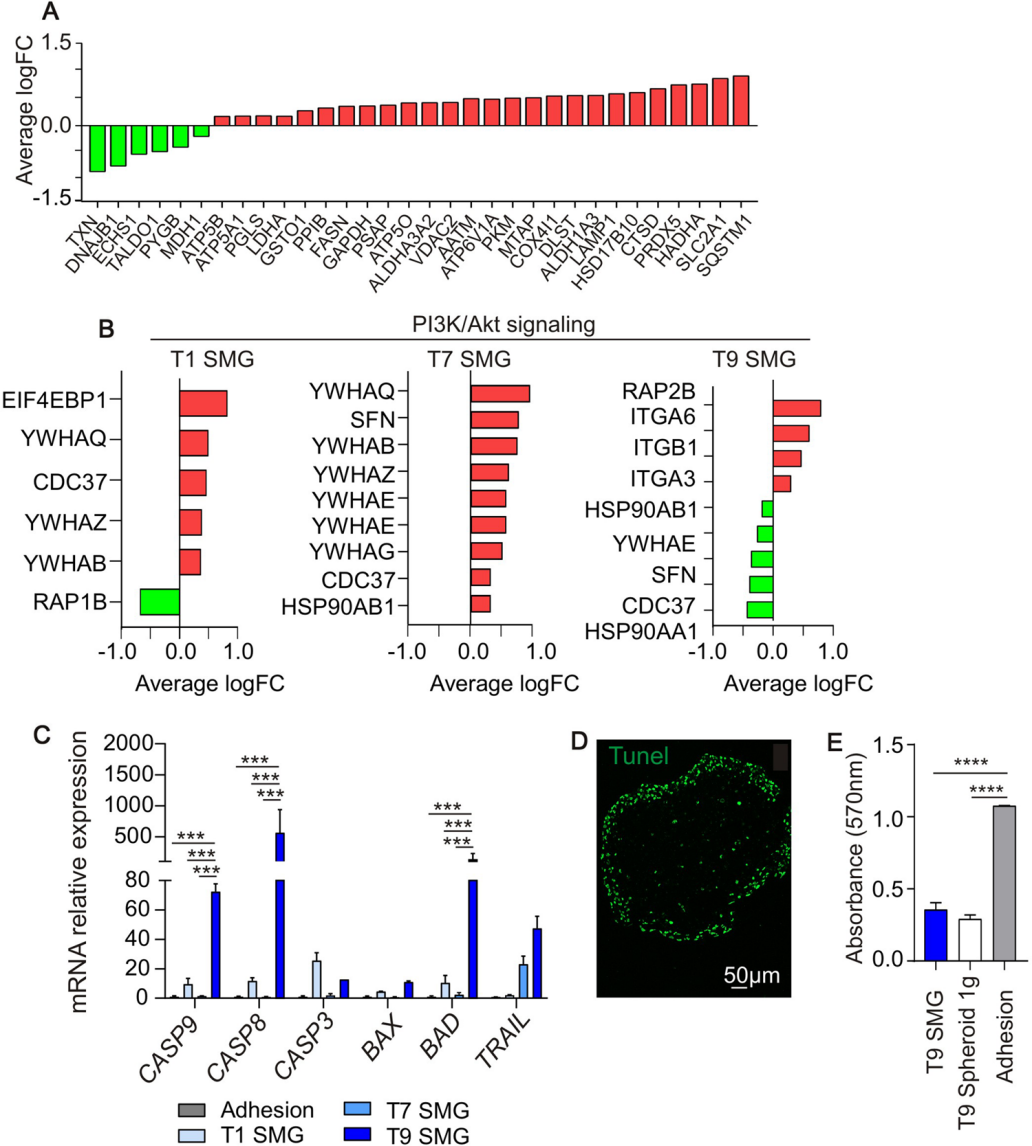
Simulated microgravity-mediated tumor cell reprogramming. **A** Average logarithmic fold change of proteins involved in metabolic-related pathways evaluated in PaCa-44 cells maintained in SMG for 9 days; in green proteins downregulated while in red proteins upregulated compared to adhesion culture conditions. **B** Average logarithmic fold change of proteins involved in PI3K/Akt signaling evaluated in PaCa-44 cells maintained in SMG for 1, 7 or 9 days (T1, T7 and T9 SMG, respectively); in green proteins downregulated while in red proteins upregulated compared to adhesion culture conditions. **C** qRT-PCR analysis of pro and anti-apoptotic genes in PaCa-44 cells maintained in normal culture condition (Adhesion) or in SMG for 1, 7 or 9 days (T1, T7 and T9 SMG, respectively). Data are expressed as mean ± sem of n = 3 replicates. *0.05; ***0.0002; **** < 0.0001. **D** One representative picture of immunofluorescence analysis for apoptosis (Tunel expression) on spheroids obtained on the 9th day in SMG. Scale bar 50 μm. **E** MTT assay in PaCa-44 cells maintained in normal adhesion culture condition (Adhesion), spheroid-forming condition (T9 Spheroid 1 g) or SMG (T9 SMG) for 9 days. Data are expressed as mean ± sem of *n* = 6 replicates

At 9 days of SMG exposure, we observed upregulation of several proteins involved in metabolic-related pathways such as autophagy (LAMP1, SQSTM1), mitochondrial activity (ATP5B, ATP5A1, ATP5P0, VDAC2, ATP6V1A, COX4I1, HSD17B10, HADHA, PRDX5), TCA cycle (DLST) and Pentose phosphate pathway (PGLS, Fig. 5A). Other pathways are involved in the glucose fate decision, including the phosphatidylinositol‐3‐kinase/Akt (PI3k/Akt) pathway, which is also a key regulator of apoptosis and cell proliferation. In our setting, we observed upregulation of PI3K/Akt signaling already on the 1^st^ and the 7^th^ day of exposure to SMG (Fig. 5B). Interestingly, after 9 days in SMG the pathway was downregulated (Fig. 5B, T9 SMG). These results, together with the upregulation of proteins involved in autophagy, suggest the cell capability of activating alternative survival mechanisms. The crosstalk between apoptosis, which invariably leads to cell death, and autophagy, which has pro-survival functions is complex. To clarify this aspect, we evaluated, at the different time points of SMG exposure, key genes of the apoptotic signaling. qRT-PCR analysis indicated a higher expression after 9 days of all the pro-apoptotic genes evaluated (i.e. Caspase 9,8,3, Bax, Bad and Trail, Fig. 5C). Tunel staining on spheroids collected on the 9th day in SMG showed apoptotic signals on the outer membrane of the structure (Fig. 5D) suggesting an external layer of apoptotic cells. Accordingly, MTT assay demonstrated a lower signal on cells maintained for 9 days in spheroid-forming culture condition both at 1 g and in SMG, compared to adhesion condition (Fig. 5E).

## Discussion

Understanding the impact of altered gravity on tumor cells represents the starting point for the development of novel anti-cancer therapies. We evaluated the impact of SMG to PDAC cells (PaCa-44) performing a comprehensive proteomic, lipidomic and transcriptomic analysis at different time points (1, 7 and 9 days); the major results have been confirmed in other two PDAC cell lines: CFPAC-1 and AsPC-1. Our aim was to elucidate the time-dependent modification induced by the alteration of the gravity force. Our data clearly indicated that SMG impacts in a time-dependent manner on the structural, metabolic and migratory profile of tumor cells selecting an aggressive subset endowed with stem-like features.

**Fig. 6:**
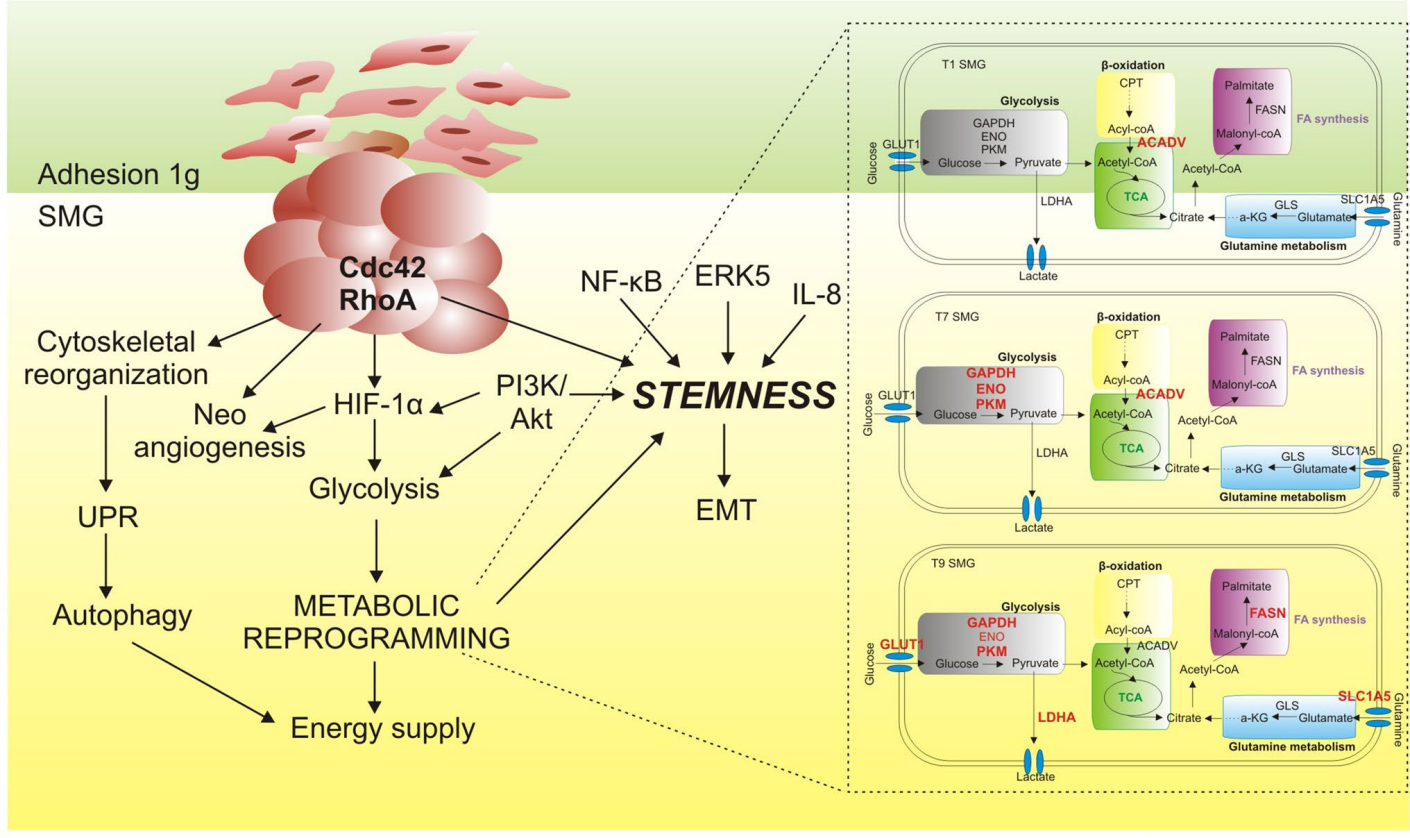
Schematic illustration of the effects of simulated microgravity (SMG) on PaCa-44 cells. Under SMG, the cells leave their adhesion elongated shape and aggregate in 3D spheroidal structures. Indeed, SMG impacts on cellular morphology through the modulation of proteins involved in Cdc42 and RhoA signaling that in turn affect cytoskeletal organization, neo-angiogenesis and stemness. Cytoskeletal reorganization impairs cell biological processes and induces cancer cells to activate survival mechanisms, among which the unfolded protein response (UPR) pathway, which supports autophagy to prevent cell death. The morphological rearrangement occurring during SMG additionally induces metabolic reprogramming orchestrated by the activation of HIF-1α and PI3K/Akt pathways, which pave the way for glucose metabolism upregulation. A more active glycolysis, compared to adhesion condition, is involved in the regulation of proliferation, metastasization, and aggressiveness. These metabolic changes, together with activation of PI3K/Akt/NF-κB/ERK5/IL-8 signaling axis induce the upregulation of stem-associated proteins and expansion of the CSC subset that supports neoplastic progression through EMT

Dissecting cell alterations induced by SMG, we speculate that in the first 24 h, to survive SMG exposure, cells collect energy in form of ATP activating the degradation of long FAs through β-oxidation in the peroxisome. This is suggested by acylcarnitine decrease and consequent impairment of mitochondrial β-oxidation together with the upregulation of acyl-CoA dehydrogenase very long (ACADV). Altogether these observations, and the fact that FAs may enter the peroxisome via ABC carrier system rather than the carnitine shuttle, suggest that there is involvement of peroxisomes in very long FA degradation rather than the classical mitochondrial β-oxidation pathway in the early days of SMG exposure. In this way, the acetyl-CoA produced by peroxisomes is used by the mitochondrial TCA cycle to support energy consumption due to cell respiration. Indeed, at this time point, the spheroids present small dimensions and are mainly composed of aggregated cells. The upregulation at 1 day of RhoA and Cdc42 signaling contributes to dynamic actin rearrangements required for cell migration which became more evident at longer time points of SMG exposure. Indeed, Cdc42 promotes cell protrusion with formation of a leading edge where a localized actin polymerization moves forward to the membrane in filopodia and lamellipodia, generating the locomotive force. On the other hand, RhoA regulates the assembly of actomyosin filaments whose contractile force induces the retraction of the cell tail [25]. The prolonged exposure (7 days) to SMG triggers actin upregulation, cytoskeleton rearrangement and deep cellular metabolic alterations to overcome the hypoxic-core formation within the spheroidal structure. Cells upregulate glycolytic enzymes (GAPDH, ENO and PKM) in order to use glucose as energy substrate in an anaerobic manner (Fig. 6). The cellular machinery is involved in the synthesis of new phospholipids (as indicated by the increase in total LPCs, LPEs and in particular species of PCs and PEs) and FAs, to sustain the formation of novel membrane lipids. We observed an increase in total FAs, comprising arachidonic acid. It has been demonstrated that PI3K/mTOR/PKC axis induces calcium-dependent phospholipase A2 (cPLA2) involved in the release of arachidonic acid, oleic acid (FA 18:1) and other bioactive lipids (such as eicosanoids) [26]. Remarkably, arachidonic acid and derived eicosanoids may have a role in CSC proliferation and be involved in the aggressive phenotype of CSCs [27, 28]. However, both the pathways sustaining stemness (i.e. IL-8 and NF-κB) and the CSC markers are not upregulated. Only at a longer time point, the selection of a stem-enriched and more aggressive subset of cells is significant. Indeed, after 9 days of SMG exposure we observed upregulation of IL-8 and NF-κB pathways that promoted expression of cell adhesion molecules and proteins involved in the invasion process (e.g. matrix metalloproteinases), characteristic of cancer stemness [29]. Moreover, NF-κB is an upstream modulator of cyclooxygenase-2 (COX-2) acting as a procarcinogenenic enzyme. COX-2 can promote tumor growth and progression activating mechanisms including immune evasion and increased invasiveness through EMT processes [29]. Indeed, on the 9th day we observed higher expression of all the evaluated mesenchymal markers and higher cell migration capability, suggesting the complete transformation towards a more stem and aggressive phenotype induced by SMG exposure, as indicated also by upregulation of EpCAM and other stemness-associated markers (Nanog, Sox2 and Oct4). NF-kB regulation is integrated in a very complex network of molecular and metabolic pathways [30, 31]. Indeed, CSCs are endowed with a more active glycolysis that leads to a higher pyruvate production, which in turn fuels ATP synthesis involved in the regulation of metastasizing and aggressiveness. In line, 9 days after SMG exposure, cells undergo a profound membrane remodeling through the synthesis of PCs from LPCs and FAs. It has been shown that PCs work as oncogenic signals [32] triggering cell proliferation and tumor growth. At this time point, we observed a reduction in total LPCs and LPEs as well as FsA. The LPCs produced at 7 days could be used together with neosynthesized FAs (such as palmitate) as supported by FASN upregulation and ECHS1 downregulation. In addition, to synthetize FAs, cells could use acetyl-CoA produced by glycolysis (GLUT1 and the other glycolytic enzyme are upregulated at this time point) and by glutamine metabolism (upregulation of SLC1A5). The surplus of pyruvate produced by glycolysis is extruded from the cells through LDHA transporter in the form of lactate favoring the acidification of the extracellular milieu, supporting the aggressive and migratory phenotype of the cells. Moreover, enhanced glucose uptake may also sustain the production of NADPH by the pentose phosphate pathway, a glycolytic flux shunt, as indicated by upregulation of PGLS. In turn, NADPH is the essential coenzyme for FAs neosynthesis and consequent formation of TGs, which may be used for either energy starvation (lipid droplets) or for membrane biosynthesis.

All together, these results describe time by time cell transformation toward a more stem and aggressive phenotype induced by SMG exposure. This cell rearrangement may also affect tumour cells susceptibility to drugs, thus future works will focus on the evaluation of pancreatic cancer cells response to anti neoplastic drugs under simulated microgravity.

## Supplementary Information

Below is the link to the electronic supplementary material.

Supplementary file1 (DOCX 450 KB)

## Data Availability

The mass spectrometry proteomics data, dataset identifier PXD028708, have been deposited to the ProteomeXchange Consortium via the PRIDE partner repository (https://www.ebi.ac.uk/pride/archive/). Dataset identifier proteomic analysis: PXD028708. Reviewer account details: Username: reviewer_pxd028708@ebi.ac.uk; Password**:** fId4pe3W.

## Abbreviations

AA: Arachidonic acid
CSC: Cancer stem cell
EMT: Epithelial-to-mesenchymal transition
FA: Fatty acid
PC: Phosphatidylcholine
PDAC: Pancreatic ductal adenocarcinoma
PE: Phosphatidylethanolamine
LPC: Lysophosphatidylcholine
LPE: Lysophosphatidylethanolamine
ROS: Reactive oxygen species
RPM: Random Positioning Machine
SMG: Simulated microgravity
TCA: Tricarboxylic acid
TG: Triglyceride/triacylglycerol
