# Supplementary material for: Prolonged exposure to simulated microgravity promotes stemness impairing morphological, metabolic and migratory profile of pancreatic cancer cells: a comprehensive proteomic, lipidomic and transcriptomic analysis
Source: Cell Mol Life Sci. 2022 Apr 7;79(5):226. doi: 10.1007/s00018-022-04243-z (PMC8990939; doi:10.1007/s00018-022-04243-z)
Supplement: Supplementary file 1 — Supplementary file1 (DOCX 450 KB) [file 18_2022_4243_MOESM1_ESM.docx]

**SUPPLEMENTARY INFORMATION**


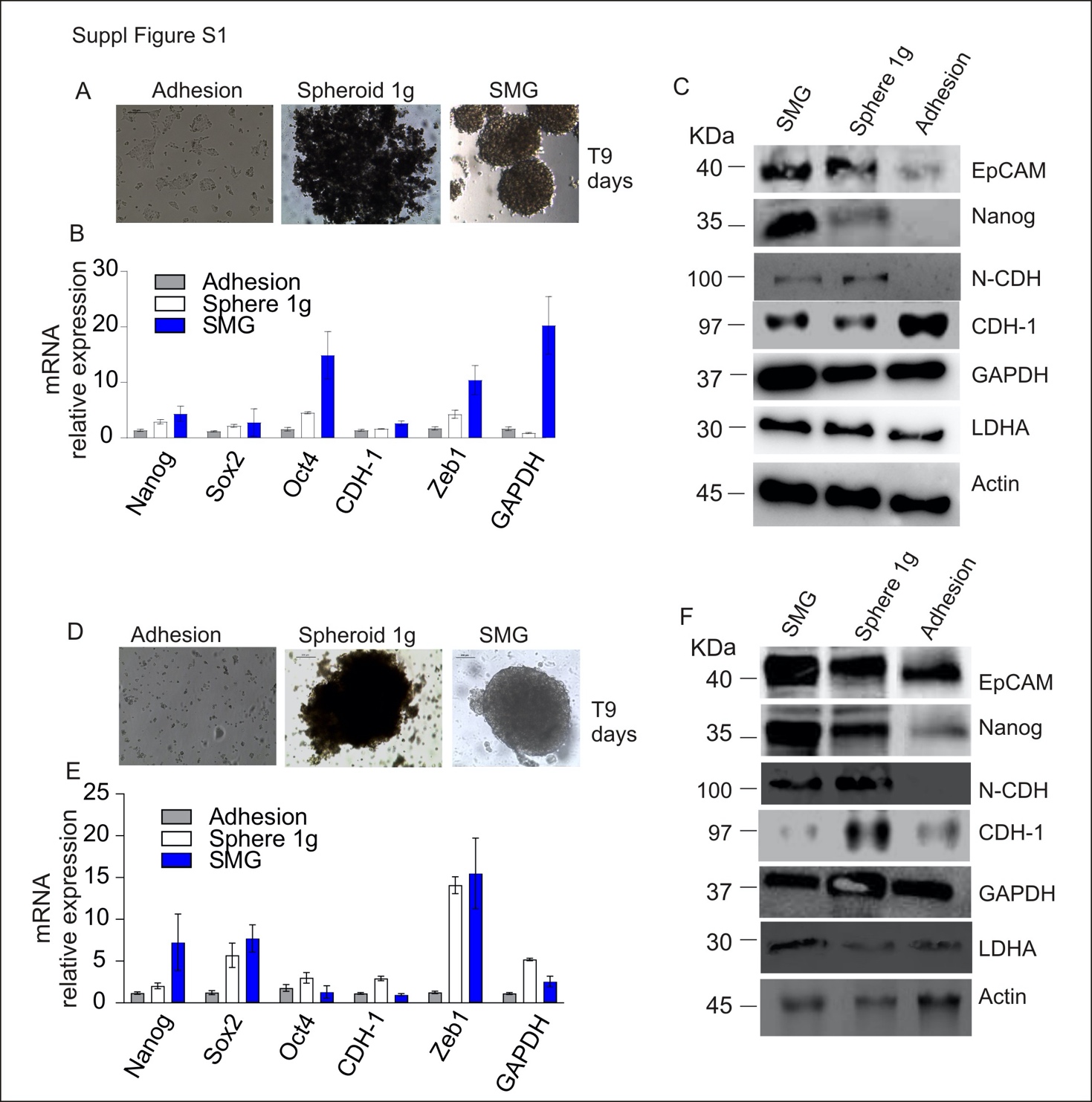


**Supplementary Figure S1: Simulated microgravity exposure induces morphological rearrangement, promotes stemness and migratory capability, and impairs cell metabolism in CFPAC-1 and AsPC-1 cells.**

**A)** CFPAC-1 cells were maintained for 9 days in normal adhesion culture condition (Adhesion), spheroid-forming at normal gravity (Spheroid 1 g) or simulated microgravity (SMG). One representative picture for each condition (Magnification 5x). **B)** qRT-PCR analysis of stemness markers (Nanog, Sox2 and Oct4), EMT-associated markers (E-cadherin and Zeb-1) and GAPDH in CFPAC-1 cells maintained in normal culture condition (Adhesion), spheroid-forming at normal gravity (Spheroid 1 g) or in simulated microgravity (SMG) for 9 days. Data are expressed as mean±sd of n=2 replicates. **C)** Western blot analysis of stemness markers (EpCAM and Nanog), EMT-associated markers E-cadherin (CDH-1) and N-cadherin (N-CDH), metabolic components (GAPDH and LDHA) and Actin in CFPAC-1 cells maintained in normal culture condition (Adhesion), spheroid-forming culture condition at normal gravity (Spheroid 1 g) or in simulated microgravity (SMG) for 9 days.

**D)** Representative picture of AsPC-1 cells maintained for 9 days in normal adhesion culture condition (Adhesion), spheroid-forming at normal gravity (Spheroid 1 g) or simulated microgravity (SMG). Magnification 5x. **E)** qRT-PCR analysis of stemness markers (Nanog, Sox2 and Oct4), EMT-associated markers (E-cadherin and Zeb-1) and GAPDH in AsPC-1 cells maintained in normal culture condition (Adhesion), spheroid-forming at normal gravity (Spheroid 1 g) or in simulated microgravity (SMG) for 9 days. Data are expressed as mean±sd of n=2 replicates. **F)** Western blot analysis of stemness markers (EpCAM and Nanog), EMT-associated markers E-cadherin (CDH-1) and N-cadherin (N-CDH), metabolic components (GAPDH and LDHA) and Actin in AsPC-1 cells maintained in normal culture condition (Adhesion), spheroid-forming culture condition at normal gravity (Spheroid 1 g) or in simulated microgravity (SMG) for 9 days.


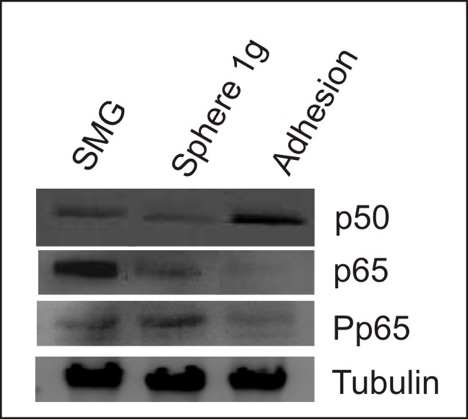


**Supplementary Figure S2. Simulated microgravity exposure triggers NF-kB pathway activation.**

Western blot analysis of p50, p65 and phospo-p65 in PaCa-44 cells maintained in normal culture condition (Adhesion), spheroid-forming condition at 1g (Spheroid 1g) or in simulated microgravity (SMG) for 9 days; Tubulin is used as housekeeping
